# Effects of Dietary Multi-Carbohydrase and Phytase Complex Supplementation on Nutrient Digestibility, Bone Mineralization and Puberty Onset in Gilts

**DOI:** 10.3390/ani15040462

**Published:** 2025-02-07

**Authors:** Fangyuan Chen, Rui Zhou, Lianpeng Zhao, Lingjie Huang, Yong Zhuo, Shengyu Xu, Yan Lin, Lianqiang Che, Bin Feng, De Wu, Zhengfeng Fang

**Affiliations:** 1 Key Laboratory for Animal Disease Resistance Nutrition of the Ministry of Education, Animal Nutrition Institute, Sichuan Agricultural University, Chengdu 611130, China; c2023fangyuan@163.com (F.C.); rui1zhou@163.com (R.Z.); zlp9426@163.com (L.Z.); 71424@sicau.edu.cn (L.H.); zhuoyong@sicau.edu.cn (Y.Z.); shengyuxu@sicau.edu.cn (S.X.); linyan@sicau.edu.cn (Y.L.); chelianqiang@sicau.edu.cn (L.C.); fengbin@sicau.edu.cn (B.F.); wude@sicau.edu.cn (D.W.); 2 College of Food Science, Sichuan Agricultural University, Ya’an 611130, China

**Keywords:** nutrient availability, bone biomarker, bone strength, enzymatic complex, reproductive onset, gilt

## Abstract

The study was conducted to determine the effects of multi-carbohydrase and phytase complex (MCPC) supplementation in standard and commercial diets on growth performance, nutrient digestibility, bone mineralization, blood biochemical parameters and puberty onset in gilts. A total of 144 healthy gilts (Duroc × (Landrace × York)) were assigned randomly to four treatments (*n* = 36), with 10 replicate pens (6 pens, each containing 4 gilts, and 4 pens, each containing 3 gilts). The trial consisted of two phases (phase 1: 70–100 kg; phase 2: 100–140 kg). The commercial diet (COM) had 33% higher calcium (Ca) and phosphorus (P) levels than the standard diet (CON) across all phases. The four treatment diets were as follows: CON (phase 1: 0.56 g/kg Ca and 0.49 g/kg P; phase 2: 0.49 g/kg Ca and 0.45 g/kg P), COM (phase 1: 0.75 g/kg Ca and 0.65 g/kg P; phase 2: 0.65 g/kg Ca and 0.60 g/kg P), CON + MCPC and COM + MCPC, where both the CON and COM diets were supplemented with 100 mg/kg of MCPC, respectively. The commercial diet significantly (*p* < 0.05) increased the total number of follicles and the number of follicles < 4 mm and tended to reduce (*p* = 0.07) the age at puberty compared to the standard diet. Besides, MCPC supplementation improved the apparent total-tract digestibility (ATTD) of Ca (*p* < 0.05), P (*p* < 0.05) and Ash (*p* = 0.07) in gilts during phase 1, compared to the basal diets without MCPC supplementation. Additionally, MCPC supplementation significantly elevated serum creatinine (CREA, *p* < 0.05) concentrations and had a tendency to increase serum Ca (*p* = 0.07) concentrations in gilts. Specifically, COM + MCPC supplementation significantly increased the osteocalcin (OCN) concentration compared with the COM treatment. Moreover, dietary MCPC supplementation significantly improved the bone strength (*p* < 0.05) compared to the basal diets without MCPC supplementation. In conclusion, dietary MCPC supplementation increased the ATTD of Ca and P in gilts, while also improving bone strength. This improvement not only extends the reproductive lifespan of sows, but it also allows for reduced supplementation levels of Ca and P in the dietary formula.

## Simple Summary

Anti-nutritional factors such as non-starch polysaccharides (NSP) and phytic acid hinder the absorption of calcium (Ca) and phosphorus (P). However, Ca and P are important nutrients for pig growth, development and skeletal mineralization processes. Exogenous enzymes are widely used in pig production to address these related issues. The study was conducted to determine the effects of multi-carbohydrase and phytase complex supplementation on growth performance, nutrient digestibility, bone mineralization and puberty onset in gilts. The results showed that dietary MCPC supplementation increased the ATTD of Ca and P in gilts, thereby reducing their environmental excretion and improving bone strength. This improvement not only helps decrease the incidence of limb and foot disorders in sows and extends their reproductive lifespan, but it also allows for lower supplemental levels of Ca and P in the dietary formula.

## 1. Introduction

The current global shortage of feed resources makes it crucial to optimize feed formulations for economic efficiency and environmental sustainability, as blindly increasing dietary nutrient content reduces nutrient utilization and wastes resources. In addition, feeds contain many anti-nutritional factors such as non-starch polysaccharides (NSP) and phytic acid. NSP can increase the viscosity of intestinal contents and interfere with nutrient absorption, reducing enzyme activity, which ultimately lowers digestibility and nutrient absorption efficiency, negatively impacting growth performance and feed utilization [1]. Furthermore, phytic acid binds to minerals such as calcium (Ca) and phosphorus (P) and proteins to form indigestible complexes, which negatively affect the digestibility of nutrients [2,3,4]. Approximately 60–80% of the P in feed is in the form of phytate phosphorus, but pigs have limited ability to release P from phytic acid in the digestive tract [2,5]. Moreover, pigs lack corresponding endogenous enzymes to degrade NSPs and phytic acid, further reducing the nutrient utilization efficiency, especially for Ca and P.

Several studies have evaluated the importance of Ca and P as essential nutrients for pig growth, development and skeletal mineralization processes [6,7]. Early-life deficiencies of Ca and P in gilts affect their reproductive performance throughout life, resulting in decreased sex hormone levels, defects in follicular development and corpus luteum formation, which contribute to delayed puberty, and other reproductive disorders, as well as higher infertility-related culling rates in older gilts [8,9]. Furthermore, approximately 96–99% Ca and 60–80% P are stored in bone in the body [10]. Gilts have higher requirements for Ca, P, selenium, copper, and zinc compared to growing-finishing pigs. However, gilts often experience negative mineral balance throughout their reproductive cycle. This is particularly concerning, as foot and leg problems in sows have long been recognized as major contributors to postpartum paralysis, especially in modern hyper-prolific sows [11]. Therefore, ensuring adequate Ca and P intake is essential for maintaining reproductive health and bone mineralization in gilts. According to the nutrient requirements of swine (NRC, 2012), the recommended Ca and total phosphorus (TP) intake for gilts at different stages (under ad libitum feeding) are as follows: 75–100 kg body weight (BW), Ca: 0.56% and TP: 0.49%; 100–135 kg BW, Ca: 0.49% and TP: 0.45%. However, in practical pig production, there is often a tendency to add excessive amounts of inorganic Ca and P to meet these challenges. This approach, however, further reduces nutrient utilization efficiency and increases the costs associated with feed and waste management [12]. Therefore, it has become increasingly important to explore appropriate nutritional strategies that balance optimal Ca and P levels in gilts, while also addressing the impact of anti-nutritional factors that affect the release of Ca, P and other nutrients. These strategies aim to improve production performance, nutrient utilization efficiency, reduce feed costs, and to minimize the waste of Ca and P resources.

Exogenous enzymes are widely used in pig production to solve the above-related problems. Studies have shown that dietary phytase or NSP enzyme supplementation improved the digestibility and bioavailability of nutrients, enhanced growth performance in pigs and reduced undigested nutrient emissions in manure [3,13,14,15]. Moreover, dietary phytase supplementation could hydrolyze phytate to release Ca and P, which improved bone mineral density, P content and the structural density of pigs [2,16]. However, limited data existed on the precise utilization of phytase in sow diets. Whether enzyme supplementation promoted the release of nutrients such as Ca and P, typically bound by anti-nutritional factors, and thus improved sow performance remained an area for further exploration, especially during high Ca and P demand phases in gilts. Moreover, due to the specificity of enzymes to their substrates, single enzyme preparations have limited effectiveness in improving the digestibility of complex dietary composition. Therefore, multi-enzyme combinations are more efficient and economically valuable than single enzymes [17,18]. Nevertheless, there is a lack of information regarding the effects of dietary phytase or NSP enzyme supplementation (MCPC: phytase, xylanase, β-glucanase and a-arabinofuranosidase) on bone metabolism and puberty onset in gilts. Our recent study in commercial pigs demonstrated the positive role of phytase-based enzyme complex in increasing the availability of Ca and P [19], which leads us to hypothesize that dietary MCPC supplementation enhances the release of nutrients typically bound by anti-nutritional factors, increasing the apparent total tract digestibility (ATTD) of Ca and P in gilts, and thereby reducing their environmental excretion, improving bone strength and puberty onset. Therefore, to verify this hypothesis, this study was based on dietary anti-nutritional factors, such as NSP. The core of the experimental design was to investigate the interaction between varying Ca and P levels and MCPC supplementation. This approach aimed to reduce the reliance on exogenous Ca and P by improving nutrient digestibility and bone mineralization in gilts, thereby enhancing the long-term reproductive performance of sows.

## 2. Materials and Methods

### 2.1. Experimental Design and Diets

A total of 144 healthy gilts (Duroc × (Landrace × York)), selected from one weaning batch, with an initial age of 130 days and mean BW of approximately 75 kg, were included in the study. All gilts were assigned to four treatments and randomly allocated to one of the four diets: CON treatment (standard diet); COM treatment (commercial diet); CON + MCPC and COM + MCPC treatments (standard and commercial diets supplemented with 100 mg/kg MCPC). Each treatment consisted of 10 replicate pens: 6 pens, each containing 4 gilts, and 4 pens, each containing 3 gilts. This grouping was consistent throughout the entire experimental phase, with gilts in each replicate having similar BW and backfat thickness (BF). The MCPC supplement was designed to provide the following enzyme activities per kg of diet: 1800 U of xylanase, 1244 U of beta-glucanase, 6600 U of a-arabinofuranosidase and 1000 FTU of phytase. MCPC was supplied by ADISSEO (Paris, France). The entire experiment was divided into two phases based on BW (phase 1: 75 to 100 kg, phase 2: 100 to 140 kg).

In each experimental phase, all diets were formulated according to the NRC (2012) recommendations to meet the nutritional requirements of gilts (Table 1). The CON and COM diets differed in calcium (Ca) and total phosphorus (TP) levels (Table 2). Specifically, for the CON diet, Ca and TP levels were 0.56% and 0.49% in phase 1, and 0.49% and 0.45% in phase 2, respectively. For the COM diet, Ca and TP levels were 0.75% and 0.65% in phase 1, and 0.65% and 0.60% in phase 2, respectively.

Gilts were subjected to restricted feeding, receiving individual meals twice a day, with an average intake of 2.5 kg/d during phase 1 and 2.8 kg/d during phase 2. All gilts had free access to water throughout the experiment. Feed consumption for each pen was recorded weekly. At the end of each phase, the total BW of each pen of gilts was measured after a 12 h fasting period. After reaching 5 months of age, all gilts were exposed to mature boars for puberty induction twice daily (at 8:00 and 16:00). Estrous was detected by only one experienced stockperson based on behavior and vulvar characteristics. Estrus performance was recorded, including estrus rate, age at first estrus, BW and BF at the time of first estrus. All gilts were purchased from Sichuan Tieqi Lishi Industry Co. Ltd (Chengdu, China), and the experiment was conducted at the Research Farm of the Animal Nutrition Institute, Sichuan Agricultural University (Ya’an, China), throughout the entire experimental period.

### 2.2. Sample Collection and Measurements

At the end of phase 1, fresh fecal samples of gilts (*n* = 10) were collected from each pen for four consecutive days and stored at −20 °C until analysis for the apparent total tract digestibility (ATTD) of crude protein (CP), gross energy (GE), dry matter (DM), Ca, P and Ash. All fecal samples were dried in a fan-forced oven at 65 °C to constant weight and then ground through a 1 mm sieve. The CP content of diet and fecal samples was analyzed using the Kjeldahl nitrogen method with a nitrogen analyzer (KJELTEC 8420, Foss, Hillerød, Denmark). GE was determined with an automatic oxygen and nitrogen calorimeter (Parr 6400, Parr Instrument Co., Ltd., Moline, IL, USA). The Ca content (Method 927.02; AOAC Int., Rockville, MD, USA, 2007) was determined using the potassium permanganate titration method. P content (Method 965.05; AOAC Int., 2007) was analyzed by colorimeter (UV-1000 ECTROPHOTO, Hanyi Technology Co., Ltd., Shanghai, China). DM and Ash contents were determined following AOAC methods 930.15 and 942.05, respectively (AOAC Int., 2007). The acid-insoluble ash in feed and feces was analyzed according to previous methods [21] and used as an in vivo marker to calculate the ATTD of nutrients using the following formula:

(1) $$ATTD\left(\%\right)=10-\frac{Indicator\;in\;feed\left(\%\right)}{Indicator\;in\;feces\left(\%\right)}\times \frac{Components\;content\;in\;feces\left(\%\right)}{Components\;content\;in\;feed\left(\%\right)}\times 100\%$$

At the end of phase 2, six gilts with body weights close to the average were selected from each treatment. After a 12 h fasting period, approximately 10 mL of blood sample of gilts (*n* = 6) was collected using the syringe method via jugular vein puncture. For serum extraction, tubes without a clot activator were used. The blood samples were centrifuged at 3000 rpm for 10 min at 4 °C to obtain serum sample, which was stored at −20 °C until analysis. Serum samples were analyzed for the following parameters: bone alkaline phosphatase (BALP), osteocalcin (OCN), Ca, P, alkaline phosphatase (ALP), creatinine (CREA), total protein, Immunoglobulin (IgM), non-esterified fatty acids (NEFA) and triglyceride (TG) concentrations. Serum BALP and OCN concentrations were measured using separate ELISA kits (Nanjing Jiancheng Bioengineering Institute, Nanjing, China), following the manufacturer’s instructions. Serum Ca, P, ALP, CREA, TP, IgM, NEFA and TG concentrations were determined at the Institute of Animal Nutrition, Sichuan Agricultural University, by using an automatic biochemical analyzer (Model 3100; Hitachi, Tokyo, Japan) according to the instructions of kits (Nanjing Jiancheng Bioengineering Institute, Nanjing, China).

After blood collection, the gilts were euthanized by electrical stunning, and the ovaries and left tibia samples were quickly collected and stored at −20 °C until analysis. After the bilateral ovaries were collected, the number of follicles on the ovaries were recorded (measured by 1–4 mm and ≥4 mm in diameter) as previously described [22]. Ovaries were used to assess the influence of puberty onset of gilts. Bone samples were analyzed for strength, Ash, Ca and P content. Bone strength of fresh bone samples was calculated by Bone Strength Tester (Wuhan Huatuo Measurement Technology Co., Ltd., Wuhan, China). After bone strength measurement, bones were dried and soaked in petroleum ether under a chemical hood for 72 h to remove the remaining marrow and fat. The fat-free bones were dried in an oven at 100 °C for 24 h to measure the Ca, P and ash contents [19].

### 2.3. Statistical Analysis

All data were analyzed by the two-way ANOVA procedure using the MIXED procedure of SAS 9.4 (SAS Institute, Inc, Cary, NC, USA) in a complete randomized design using the following models:

Y_ijk_ = µ+ α_i_ + β_j_ + (αβ)_ij_ + e_ijk_ (2)

where Y_ijk_ is an observed trait, µ is the population mean, α_i_ is the fixed effect of the diet (i = standard diet or commercial diet), β_j_ is the fixed effect of the enzyme (MCPC) (j = with enzyme, or without enzyme), (αβ)_ij_ is the interaction between the diet and MCPC, and e_ijk_ is the residual, which was assumed to be normally distributed and to have variance homogeneity. Differences between means were determined using Tukey’s test. The individual pig or pen served as the experimental unit for all analyses. The correlation analysis of bone parameters (bone strength, bone Ca and bone P) and serum parameters (OCN, BALP, Ca, P and ALP) was performed with Person correlation analysis. The data are presented as means with SEM, where *p* < 0.05 was considered to be statistically significant.

## 3. Results

### 3.1. Effects of Dietary MCPC Supplementation on the Growth Performance of Gilts

Table 3 shows the effects of MCPC supplementation in standard and commercial diets on the growth performance of gilts. The BW, backfat thickness, average daily gain (ADG) and feed conversion rate (FCR) of gilts were not significantly affected (*p* > 0.10) by the diet, MCPC supplementation, or the interaction between the diet and MCPC, regardless of whether the measurements were taken during phase 1 or phase 2.

### 3.2. Effects of Dietary MCPC Supplementation on the Follicle Development and Puberty Onset of Gilts

As shown in Table 4, the number of total follicles and the number of follicles (<4 mm) were significantly affected (*p* < 0.05) by diet and MCPC supplementation. Gilts fed the commercial diet exhibited an increased (*p* < 0.05) number of total follicles and follicles (<4 mm) and had a tendency (*p* = 0.07) to reduce the age at puberty compared to those fed the standard diet. Additionally, dietary MCPC supplementation significantly reduced (*p* < 0.05) the number of total follicles and follicles (<4 mm) compared to basal diets. However, the interaction between diet and MCPC supplementation did not affect BW and backfat thickness at puberty, estrus rate, the number of large follicles (≥4 mm) and the ratio of large follicles (≥4 mm).

### 3.3. Effects of Dietary MCPC Supplementation on the Nutrient Digestibility of Gilts

**Figure 1:**
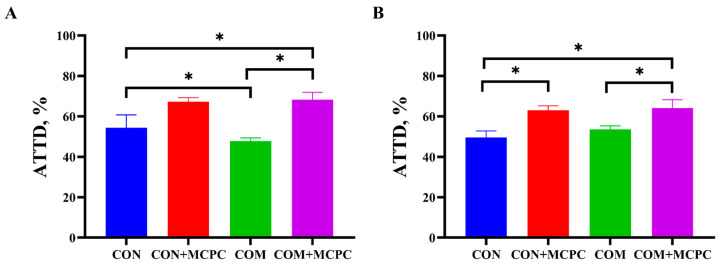
Effects of supplemental MCPC in standard diet and commercial diet on apparent total-tract digestibility of nutrients. (**A**), ATTD of Ca; (**B**), ATTD of P; CON = standard diet; COM = commercial diet; CON + MCPC = standard diet + multi-enzymes; COM + MCPC = commercial diet + multi-enzymes; ATTD = apparent total tract digestibility; Statistical significance between groups is indicated by * (*p* < 0.05, by Tukey’s test), *n* = 10.

Table 5 shows the effects of dietary MCPC supplementation on the ATTD of GE, CP, Ca, P, DM and Ash in gilts during phase 1 (75–100 kg). The ATTD of Ca in gilts was significantly affected (*p* < 0.05) by the interaction between diet and MCPC supplementation (Figure 1). Compared to standard diets, commercial diets significantly decreased (*p* < 0.05) the ATTD of Ca in gilts. However, dietary MCPC supplementation significantly (*p* < 0.05) improved the ATTD of both Ca and P, and had a tendency (*p* = 0.07) to elevate the ATTD of Ash in gilts compared to the basal diets.

### 3.4. Effects of Dietary MCPC Supplementation on the Blood Biochemical Parameters of Gilts

Dietary MCPC supplementation significantly improved serum CREA (*p* < 0.05) concentrations and had a tendency to increase serum Ca (*p* = 0.07) concentrations in gilts compared to the basal diets (Table 6). Additionally, serum OCN concentrations were significantly (*p* < 0.05) affected by the interaction between diet and MCPC supplementation. Gilts receiving the commercial diet with MCPC supplementation showed a significant increase (*p* < 0.05) in serum OCN concentration compared to those on the COM diet alone. Furthermore, although the interaction between diet and MCPC supplementation did not significantly affect the serum ALP and CREA contents of gilts, but the CON + MCPC treatment showed a 40% increase in serum ALP and an 18% increase in CREA concentrations compared to the CON treatment.

### 3.5. Effects of Dietary MCPC Supplementation on the Bone Mineralization of Gilts

Dietary MCPC supplementation significantly improved (*p* < 0.05) tibia bone strength in gilts, resulting in a 23.65% increase compared to diets without MCPC (Table 7). In the control diets, the bone strength of gilts increased by 29.6% following MCPC supplementation. In the commercial diets, there was an 18.4% increase in the bone strength of gilts with MCPC supplementation. However, bone strength and Ca, P and Ash percentages in the tibia of gilts were not significantly affected by the interaction of diet and MCPC supplementation.

### 3.6. Effects of MCPC Supplementation on Correlation of Bone Quality Parameters and Serum Parameters in Gilts

**Figure 2:**
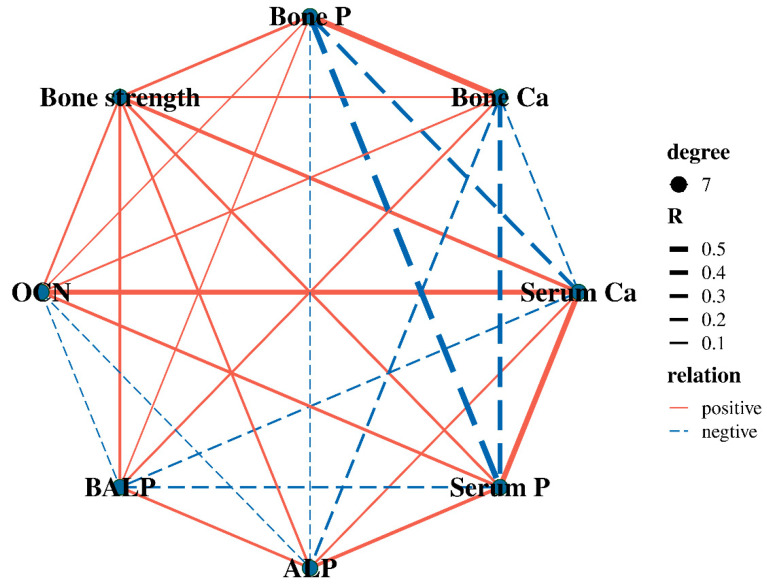
The correlation analysis between bone parameters and serum markers. Ca = calcium; P = phosphorus; ALP = alkaline phosphatase, BALP = Bone alkaline phosphatase; OCN = osteocalcin. *n* = 6; Serum OCN was positively correlated with serum Ca (*p* = 0.03), and serum Ca and P levels were positively correlated (*p* = 0.01); Bone Ca was positively correlated with bone P (*p* = 0.005); Serum P was negatively correlated with Bone P (*p* = 0.006).

As shown in Figure 2, serum OCN levels were significantly positively correlated with serum Ca content in gilts (*p* < 0.05), and there was also a significant positive correlation between serum Ca and P levels (*p* < 0.05). Bone Ca content was positively correlated with bone P (*p* < 0.01), while bone P was negatively correlated with serum P levels (*p* < 0.01).

## 4. Discussion

Our study found no significant differences in the growth performance of gilts across the different treatments throughout the experimental phases. Research on the effects of carbohydrase and phytase supplementation in gilts is limited. Phytase breaks down phytate phosphorus, releasing inorganic P and inositol, which can improve insulin sensitivity and thus affect growth performance [23]. Some studies have shown that high doses of phytase (>1500–2000 FTU/kg) improved the growth performance of pigs [24,25,26]. However, in our study, phytase was included at a typical dose of 1000 FTU/kg, which may not have been sufficient to significantly enhance growth performance due to its limited effect on P availability. Furthermore, Varley et al. reported an interaction between dietary P levels and phytase supplementation, where phytase supplementation improved growth performance in pigs when P levels were low (1.5 g/kg). In contrast, in medium and high P diets (2–2.5 g/kg), phytase supplementation did not affect growth performance [12]. In our study, the Ca and P levels met the nutritional requirements of gilts, which may explain the lack of significant differences in growth performance. Therefore, the discrepancy with previous research findings may be due to the use of a standard phytase dosage, which, under conditions where Ca and P levels are adequate, was not sufficient to further enhance the growth performance of the replacement gilts.

Earlier entry into the breeding stage and improved estrus performance in gilts can significantly enhance conception rates and overall reproductive performance in sows [27]. Additionally, the ovulation rate and follicle quality during estrus depend on the preceding follicle development process [28]. In the present study, compared to the control diets, the commercial diets tended to shorten the age at puberty and increase both the number of total follicles and follicles (<4 mm) in gilts. The main difference between the commercial and control diets is the higher Ca and P levels in the commercial diet, while energy levels remained the same. Previous studies reported that high Ca and P levels promote estradiol (E2) secretion [29,30], with estrogen playing a direct role in inducing estrus. Thus, the influence of diet on estrus age may be partly related to E2 concentrations in the body. Additionally, Calcium acts as a key second messenger in various signaling pathways, with fluctuations in intracellular Ca^2+^ concentration regulating oocyte maturation, activation, fertilization, granulosa and cumulus cell functions. These processes are essential for optimal follicular development and subsequent embryonic development [31]. Additionally, the concentrations of Ca and P in follicular fluid are closely associated with follicular development. Previous studies comparing the concentrations of various biochemical components in small, medium, and large follicular fluids have found that the Ca concentration in follicular fluid significantly increased as the follicle developed, while the P concentration showed the opposite trend [32]. Therefore, the higher levels of Ca and P in the commercial diet could facilitate an environment conducive to enhanced follicular growth and the earlier onset of puberty.

Interestingly, the distinct roles of Ca and P in follicular fluid—where Ca concentration increases and P concentration decreases as the follicle develops [32]—suggest that this inverse relationship reflects their respective roles in regulating the follicular environment and oocyte quality. In our study, dietary MCPC supplementation reduced both the number of total follicles and follicles (<4 mm) in gilts. Regardless of whether the gilts were fed the control or commercial diet, MCPC supplementation enhanced the digestion and absorption of Ca and P. This suggests that MCPC may improve the absorption of these critical minerals for reproductive function. However, excess Ca and P intake, or an imbalance in the ratio of Ca and P, can negatively impact ovarian function through hormonal feedback regulation, disrupted Ca signaling and phosphate toxicity [33,34,35]. Additionally, we speculate that in immature gilts, high levels of Ca and P may prioritize bone metabolism (resulting in significantly improved bone strength), while other tissues, such as the reproductive system, may receive lower priority. Previous studies have suggested that high-energy diets may enhance ovarian follicular development and oocyte maturation in gilts [36]. Notably, compared to growing pigs, the hindgut microbiota in gilts is more developed and has a stronger fermentation capacity. Enzymes primarily exert their enzymatic action on substrates in the foregut. In the absence of MCPC supplementation, more complex nutrients, such as resistant starch, NSP, and protein complexes, may reach the hindgut for fermentation, leading to the production of relatively higher levels of short-chain fatty acids (SCFAs) and insulin [37,38], despite no significant difference in the ATTD of GE observed in this study. Previous studies have indicated that SCFAs promote follicular development and oocyte maturation, directly or indirectly, through multiple pathways such as improving metabolism, modulating endocrine function, and influencing the synthesis of sex hormones [39]. Insulin, as an endocrine factor, plays a key role in coordinating the transition from primordial to primary follicles [40]. Therefore, these findings suggest that MCPC supplementation may influence the follicular development in gilts by enhancing the absorption and utilization of various nutrients in both the foregut and hindgut. However, its overall impact on follicular development requires further investigation.

Dietary cell walls contain non-starch polysaccharides (NSP), which can inhibit nutrient digestion and absorption by trapping nutrients and increasing the viscosity of chyme [41]. In the present study, there were no significant differences in the ATTD of DM, GE and CP in gilts with MCPC dietary supplementation. Similarly, a previous study reported that multi-enzyme (xylanase, β-glucanase, arabinofuranosidase and phytase) supplementation did not enhance the ATTD of DM, GE, CP and Ash in growing–finishing pigs, potentially due to variations in dietary Ca and P levels. These results suggest that nutrient digestibility is significantly influenced by the interaction between enzyme supplementation and mineral content [42]. Additionally, during the enzymatic hydrolysis of NSP, some intermediates (such as oligosaccharides or partially decomposed fibers) may be formed, which could competitively bind to digestive enzymes (like pancreatic enzymes), inhibiting their ability to degrade proteins and starch [43]. However, the impact of multi-enzyme supplementation on nutrient digestibility in pigs remains inconclusive. For instance, Zhang et al. reported that diets supplemented with enzymes (amylase, protease and xylanase) increased the ATTD of DM, CP and GE in piglets [44]. Furthermore, in this study, dietary MCPC supplementation improved the ATTD of Ca from 54.06% to 67.74% and the ATTD of P from 51.52% to 63.57%, compared to diets without MCPC. This is consistent with several studies showing that dietary phytase can improve the digestibility and utilization of Ca and P, while also reducing related environmental pollution [12,45,46]. A recent study demonstrated that the phytase can increase the release of digestible phosphorus in the diet by approximately 1.5 g/kg. [47]. Moreover, the ATTD of Ca was influenced by the interaction between diet and MCPC supplementation (Figure 1). Compared to the CON treatment, the ATTD of Ca was significantly lower in the COM treatment. The high Ca content in the COM diet (calcium dihydrogen phosphate: 0.30% vs. 1.17%) may promote binding with phytic acid, oxalate, and other anti-nutritional factors in the acidic environment of the stomach, indicating that simply increasing mineral supplementation does not necessarily provide more bioavailable substrates but may lead to mineral wastage. Furthermore, the improvement in ATTD of Ca and P due to MCPC supplementation was more pronounced in the COM diet than in the CON diet, with the highest ATTD of Ca observed in the COM + MCPC group. This suggests that MCPC can effectively degrade dietary anti-nutritional factors and release chelated minerals for absorption.

In the present study, dietary MCPC supplementation significantly increased the serum CREA concentration in gilts, but no significant differences were observed in serum total protein levels. Proteins metabolized in the body generate CREA, which can be classified into two types: endogenous (metabolism of body proteins) and exogenous (metabolism of dietary proteins). Serum total protein is closely related to the body’s protein metabolism. Previous studies indicated that the diet supplemented with xylanase and β-glucanase improved the digestibility of amino acids in growing pigs and sows [48,49]. However, despite the CP levels being consistent across the control and commercial diets, and MCPC not significantly improving the ATTD of CP, MCPC supplementation may have increased the metabolic rate of protein. This is evidenced by a 2.7% rise in serum total protein concentration, suggesting that the elevated CREA levels could be associated with the increased secretion of endogenous proteins due to MCPC supplementation.

Pearson correlation analysis in this study demonstrated a positive correlation between serum OCN and serum Ca, as well as between serum Ca and P levels. Although dietary MCPC supplementation showed a trend toward increasing serum Ca levels, it did not significantly affect serum P levels. It is worth noting that P-sensitive receptors are mainly located in the gut and parathyroid glands [50]. The regulation of Ca and P balance in the body is mediated by multiple hormones, including parathyroid hormone, fibroblast growth factor 23, and calcitonin [7,51]. Therefore, the lack of a significant change in serum P levels may be attributed, at least in part, to the complex regulatory effects of these hormones.

Inadequate bone mineralization due to Ca and P deficiencies is associated with a significantly higher culling rate in first- and second-litter sows [52]. A previous study reported that multi-enzyme (NSP enzymes and phytase) supplementation improved bone strength in growing–finishing pigs [53]. Additionally, phytase supplementation in diets with decreased Ca and P levels increased bone strength without affecting Ca and P content in bones [54]. Similarly, our results showed that MCPC supplementation in both standard and commercial diets improved bone strength without a significant effect on the Ash, Ca and P content of bone. Bone strength is influenced by both the quantity and quality of bone. Markers such as OCN, ALP and BALP are commonly associated with bone formation [55], with OCN playing a role in bone remodeling through a negative feedback mechanism [55]. In this study, the COM + MCPC treatment showed the highest levels of OCN and the greatest bone strength compared to the COM treatment. Previous studies reported that OCN improves bone quality by aligning biological apatite parallel to the collagen fibrils [56]. Additionally, we observed a significant reduction in bone strength in the CON treatment compared to the other three treatments, despite no significant difference in serum OCN levels. These results indicate that MCPC supplementation in the control diet has an effect on improving bone strength comparable to that in the commercial diet. It is worth noting that during Ca restriction, serum OCN levels may rise due to increased bone turnover, suggesting that changes in serum OCN are linked not only to Ca supply but also to bone metabolic activity [57]. This implies that the body may maintain bone health by increasing bone turnover when Ca is in limited supply. ALP is highly expressed in mineralized tissues and increases the rate of local inorganic phosphate production [58]. Studies have shown that adjusting ALP levels through nutritional strategies can promote osteoblast maturation, thereby enhancing bone strength [59]. Moreover, BALP supports extracellular mineralization by releasing inorganic phosphate from inorganic pyrophosphate [60]. In our study, MCPC supplementation resulted in a 20.44% and 20.23% increase in serum ALP and BALP levels, respectively, which may contribute to the observed improvements in bone strength. Although there were no statistically significant differences in bone Ash, Ca and P contents, MCPC supplementation in the control diet increased these contents by 3.72%, 3.83% and 7.70%, respectively. The levels of Ash, Ca and P in the bones of gilts in the CON + MCPC treatment were comparable to those in the COM treatment, with bone strength showing a 14.30% improvement. This suggests that dietary MCPC supplementation can effectively enhance bone mineralization in gilts, potentially providing a cost-effective strategy for conserving dietary Ca and P resources.

## 5. Conclusions

Dietary MCPC supplementation increased the ATTD of Ca and P and enhanced bone strength in gilts. This improvement not only helped to extend their reproductive lifespan, but also allowed for lower supplemental levels of Ca and P in the diet formulation. The commercial diet, which contained higher levels of Ca and P, effectively promoted follicular development and accelerated puberty onset in gilts.

## Figures and Tables

**Table 1:** Ingredients and chemical compositions of basal diet (as-fed basis, %).

Ingredients, %	Phase 1 (75–100 kg)		Phase 2 (100–140 kg)	
	CON	COM	CON	COM
Corn	67.93	66.58	66.82	65.82
Soybean meal (CP 44%)	18.00	18.15	16.00	16.20
Fish meal (CP 65%)	2.00	2.00	1.00	1.00
Wheat bran	9.00	8.90	12.60	12.30
Soybean oil	1.30	1.80	2.00	2.40
L-lysine HCl (78%)	0.11	0.11	0.04	0.04
Monocalcium phosphate	0.30	1.17	0.15	0.97
Limestone	0.80	0.73	0.83	0.72
NaCl	0.30	0.30	0.30	0.30
Vitamin and mineral premix *	0.26	0.26	0.26	0.26
Nutrient level (calculated)				
Crude protein, %	16.25	16.25	15.17	15.12
Crude fat, %	4.51	4.96	5.22	5.57
Crude fiber	3.23	3.21	3.36	3.33
Ash, %	2.41	2.39	2.41	2.40
NSP,%	16.43	16.25	17.30	17.09
Net energy, kcal/kg	2484.20	2484.20	2493.28	2496.60
SID lysine, %	0.77	0.77	0.64	0.64
SID methionine, %	0.24	0.24	0.22	0.22
SID threonine, %	0.48	0.48	0.44	0.44
SID tryphophan, %	0.15	0.15	0.14	0.14
SID isoleucine, %	0.54	0.54	0.50	0.50
SID valine, %	0.61	0.60	0.56	0.56
Calcium, %	0.56	0.75	0.49	0.65
Total phosphorus, %	0.49	0.65	0.45	0.60
STTD phosphorus, %	0.29	0.39	0.22	0.34

* Provided per kg of diet for 75–100 kg and 100–140 kg phases: vitamin premix 250 g (vitamin A, 6000 IU; vitamin D_3_, 1200 IU; vitamin E, 50 mg; vitamin K_3_, 2.4 mg; vitamin B_1_, 1 mg; vitamin B_2_, 3.6 mg; vitamin B_6_, 1.8 mg; vitamin B_12_, 12.5 μg; biotin, 0.24 mg; folic acid, 2 mg; nicotinamide, 200 mg; pantothenic acid, 12.5 mg; copper, 10 mg; iron, 120 mg; manganese, 20 mg; zinc, 150 mg; iodine, 0.28 mg; selenium, 0.3 mg). SID = standardized ileal digestible; STTD = standardized total tract digestible. NSP = non-starch polysaccharides; NSP was calculated as dry matter content minus ash, crude protein, crude fat, starch and sugar content [20].

**Table 2:** Calcium and total phosphorus levels of standard and commercial diets for gilts.

Item	Phase 1		Phase 2	
	CON	COM	CON	COM
Ca, %	0.56	0.75	0.49	0.65
TP, %	0.49	0.65	0.45	0.60

CON = standard diet; COM = commercial diet; TP = total phosphorus.

**Table 3:** Effects of supplemental MCPC in standard diet and commercial diet on the growth performance of gilts.

Item	Treatment				SEM	Diet		MCPC		*p*-Value		
	CON	CON + MCPC	COM	COM + MCPC		CON	COM	−MCPC	+MCPC	Diet	MCPC	Diet × MCPC
Initial												
BW, kg	77.03	77.03	77.03	77.02	2.12	77.03	77.02	77.03	77.02	0.99	0.99	0.99
BF, mm	5.73	5.77	5.80	5.89	0.09	5.75	5.84	5.77	5.82	0.30	0.54	0.85
Phase 1												
BW, kg	102.92	102.67	103.02	103.23	1.68	102.79	103.12	102.97	102.95	0.85	0.99	0.90
BF, mm	8.97	9.04	8.93	9.02	0.33	9.01	8.97	8.95	9.03	0.92	0.80	0.98
ADG, g/d	739.76	732.62	742.74	748.81	23.04	736.19	745.77	741.25	740.71	0.68	0.96	0.78
FCR	3.26	3.30	3.23	3.22	0.10	3.28	3.23	3.25	3.26	0.60	0.89	0.78
Phase 2												
BW, kg	133.11	131.77	130.96	133.19	1.45	132.44	132.08	132.04	132.48	0.80	0.77	0.23
Backfat thickness, mm	12.44	12.54	13.03	13.10	0.41	12.49	13.06	12.74	12.82	0.17	0.84	0.97
ADG, g/d	724.47	707.36	724.21	723.05	13.04	715.91	723.63	724.33	715.21	0.56	0.49	0.54
FCR	3.52	3.61	3.52	3.53	0.07	3.57	3.53	3.52	3.57	0.53	0.44	0.54

CON = standard diet; COM = commercial diet; BW = body weight; BF = Backfat thickness; ADG = average daily gain; FCR = feed conversion rate. *n* = 10. *p* < 0.05 means significant differences among treatments (by Tukey’s test).

**Table 4:** Effects of supplemental MCPC in standard diet and commercial diet on the puberty onset of gilts.

Item	Treatment				SEM	Diet		MCPC		*p*-Value		
	CON	CON + MCPC	COM	COM + MCPC		CON	COM	−MCPC	+MCPC	Diet	MCPC	Diet × MCPC
Age at puberty, d	206.28	204.45	201.42	202.23	1.90	205.36	201.83	203.84	203.34	0.07	0.79	0.49
BF at puberty, mm	11.45	10.93	11.56	11.22	0.35	11.19	11.39	11.51	11.07	0.58	0.24	0.80
BW at puberty, kg	124.82	123.38	121.92	120.87	2.59	124.10	121.39	123.37	122.12	0.30	0.63	0.94
Estrus rate %	79.17	73.33	77.50	83.33	7.48	76.25	80.42	78.33	78.33	0.58	1.00	0.44
Number of visible follicles, *n*												
Total follicles	45.00	26.33	74.00	45.83	8.17	35.92	60.08	59.91	36.08	<0.01	<0.01	0.57
Diameter ≥ 4 mm	4.92	4.83	7.33	4.33	1.18	4.88	5.83	6.13	4.58	0.42	0.20	0.22
Diameter < 4 mm	40.58	21.50	67.00	41.50	8.56	31.04	54.25	53.79	31.5	<0.01	0.01	0.71
Diameter ≥ 4 mm ratio, %	0.14	0.26	0.15	0.11	0.05	0.20	0.13	0.14	0.19	0.19	0.39	0.11

CON = standard diet; COM = commercial diet; CON + MCPC = standard diet + multi-enzymes; COM + MCPC = commercial diet + multi-enzymes. *n* = 10 (puberty indicators) or 6 (NO. of follicles). *p* < 0.05 means significant differences among treatments.

**Table 5:** Effects of supplemental MCPC in standard diet and commercial diet on apparent total-tract digestibility of nutrients in gilts on phase 1.

Item, %	Treatment				SEM	Diet		MCPC		*p*-Value		
	CON	CON + MCPC	COM	COM + MCPC		CON	COM	−MCPC	+MCPC	Diet	MCPC	Diet × MCPC
Ash	57.54	61.14	55.58	60.76	2.30	59.34	58.17	56.56	60.59	0.62	0.07	0.74
DM	89.36	89.06	88.82	89.63	0.65	89.21	89.22	89.09	89.35	0.98	0.70	0.40
GE	89.23	88.43	89.04	89.68	0.67	88.83	89.36	89.13	89.06	0.44	0.91	0.29
CP	88.59	87.18	87.99	88.60	0.75	87.89	88.29	88.29	87.89	0.60	0.60	0.19
P	49.57	63.03	53.47	64.11	2.93	56.30	58.79	51.52	63.57	0.41	<0.01	0.64
Ca	60.36 ^b^	67.26 ^ab^	47.77 ^c^	68.21 ^a^	2.51	63.81	57.99	54.06	67.74	0.03	<0.01	0.01

CON = standard diet; COM = commercial diet; CON + MCPC = standard diet + multi-enzymes; COM + MCPC = commercial diet + multi-enzymes; DM = dry matter; GE = gross energy; CP = crude protein; P = phosphorus; Ca = calcium; *n* = 10. *p* < 0.05 means significant differences among treatments; ^a,b,c^ Means within a row with no common letters differ at *p* < 0.05 (by Tukey’s test).

**Table 6:** Effects of supplemental MCPC in standard diet and commercial diet on blood biochemical indicators of gilts.

Item	Treatment				SEM	Diet		MCPC		*p*-Value		
	CON	CON + MCPC	COM	COM + MCPC		CON	COM	−MCPC	+MCPC	Diet	MCPC	Diet × MCPC
Total protein, g/L	69.01	71.44	67.50	68.76	1.70	70.22	68.13	68.26	70.1	0.23	0.29	0.74
NEFA, mmol/L	0.06	0.07	0.04	0.02	0.02	0.07	0.03	0.05	0.04	0.16	0.73	0.43
TG, mmol/L	0.35	0.38	0.40	0.41	0.04	0.36	0.37	0.34	0.39	0.79	0.21	0.59
CREA, µmol/L	117.71	138.47	119.88	132.75	5.01	128.09	126.27	118.75	135.61	0.72	0.03	0.45
Ca, mmol/L	2.35	2.41	2.25	2.37	0.05	2.38	2.31	2.30	2.39	0.13	0.07	0.52
P, mmol/L	2.31	2.27	2.26	2.26	0.07	2.29	2.26	2.29	2.27	0.62	0.77	0.73
ALP, mmol/L	63.50	89.00	71.83	74.00	8.02	76.25	72.92	67.67	81.5	0.68	0.10	0.16
BALP, ng/mL	36.00	35.60	36.47	51.55	7.65	35.8	44.01	36.24	43.57	0.30	0.35	0.32
OCN, ng/mL	20.66 ^ab^	19.32 ^ab^	17.94 ^b^	23.02 ^a^	1.56	19.99	20.48	19.3	21.17	0.75	0.75	0.05
IgM, g/L	0.65	0.69	0.67	0.65	0.04	0.67	0.66	0.66	0.67	0.87	0.85	0.48

CON = standard diet; COM = commercial diet; CON + MCPC = standard diet + multi-enzymes; COM + MCPC = commercial diet + multi-enzymes. NEFA = non-esterified fatty acids; TG = triglyceride; CREA = creatinine; Ca = calcium; P = phosphorus; ALP = alkaline phosphatase, BALP = Bone alkaline phosphatase; OCN = osteocalcin; IGM = Immunoglobulin M; *n* = 6. *p* < 0.05 means significant differences among treatments. ^a,b^ Means within a row with no common letters differ at *p* < 0.05 (by Tukey’s test).

**Table 7:** Effects of supplemental MCPC in standard diet and commercial diet on bone strength and calcium, phosphorus and ash percentage in bone of gilts.

Item	Treatment				SEM	Diet		MCPC		*p*-Value		
	CON	CON + MCPC	COM	COM + MCPC		CON	COM	−MCPC	+MCPC	Diet	MCPC	Diet × MCPC
Bone strength, N	4207.30	5454.69	4772.40	5648.96	384.21	4830.99	5210.68	4489.85	5551.83	0.33	0.01	0.63
P, %	9.74	10.49	10.63	10.61	0.34	10.11	10.62	10.19	10.55	0.15	0.29	0.26
Ca, %	22.17	23.02	23.23	23.24	0.46	22.59	23.23	22.70	23.13	0.18	0.36	0.37
Ash, %	56.98	59.10	59.40	58.80	1.00	58.04	59.1	58.19	58.95	0.30	0.45	0.19

CON = standard diet; COM = commercial diet; CON + MCPC = standard diet + multi-enzymes; COM + MCPC = commercial diet + multi-enzymes. *n* = 6. *p* < 0.05 means significant differences among treatments.

## Data Availability

The original contributions presented in this study are included in the article. Further inquiries can be directed to the corresponding author.
